# Complete chloroplast genome sequence of *Maytenus guangxiensis* (celastraceae), a rare and critically endangered species endemic to China

**DOI:** 10.1080/23802359.2019.1710278

**Published:** 2020-01-14

**Authors:** Yancai Shi, Bingbing Liu

**Affiliations:** a Institute of Loess Plateau, Shanxi University, Taiyuan, China;; b Guangxi Institute of Botany, Guangxi Zhuang Autonomous Region and Chinese Academy of Sciences, Guilin, China

**Keywords:** Maytenus, chloroplast genome, phylogenetic analysis

## Abstract

*Maytenus guangxiensis* (Celastraceae) is a rare and critically endangered species endemic to China. Here, we first report and characterize its complete chloroplast genome sequence based on Illumina paired-end sequencing data. The complete plastid genome was 157,102 bp, which contained inverted repeats (IR) of 26,476 bp separated by a large single-copy (LSC) and a small single copy (SSC) of 85,559 bp and 18,591 bp, respectively. The cpDNA contains 130 genes, comprising 85 protein-coding genes, 37 tRNA genes and 8 rRNA genes. The overall GC content of the plastome is 37.3%. The phylogenetic analysis of 19 selected chloroplast genomes demonstrated that *M. guangxiensis* was close to the species *Catha edulis.*

*Maytenus guangxiensis* C. Y. Cheng et W. L. Sha, an evergreen shrub which belongs to the family Celastraceae, is a rare and critically endangered species endemic to China. It’s limited distributed on the Guangxi Zhuang Autonomous Region of China. *M. guangxiensis* grows on the dank limestone areas and has an important ecological and medicinal value (Lu et al. 1989). However, largely due to anthropogenic cutting and climatic environment’s change, *M. guangxiensis* is treated as rare and endangered species in China and has been registered on the China Species Red List (Fu 1991). It is thus urgent to take effective measures to conserve this critically endangered and precious species. Herein, we first reported and characterized its complete plastome based on Illumina paired-end sequencing data, which will contribute to the further studies on its genetic research and resource utilization. The annotated cp genome of *M. guangxiensis* has been deposited into GenBank with the accession number MN707924.

In this study, *M. guangxiensis* was sampled from in Guangxi Zhuang Autonomous Region of China, located at 106°49′33″ E, 22°38′48″ N. A voucher specimen (Y.-C. Shi et al. H1328) was deposited in the Guangxi Key Laboratory of Plant Conservation and Restoration Ecology in Karst Terrain, Guangxi Institute of Botany, Guangxi Zhuang Autonomous Region and Chinese Academy of Sciences, Guilin, China. The experiment procedure is as reported in Zhang et al. (2019). Around 2 Gb clean data were used for the cp genome de novo assembly by the program NOVOPlasty (Dierckxsens et al. 2017) and direct-viewing in Geneious R11 (Biomatters Ltd., Auckland, New Zealand). Annotation was performed with the program Plann (Huang and Cronk 2015) and Sequin (http://www.ncbi.nlm.nih.gov/).

The chloroplast genome of *M. guangxiensis* is a typical quadripartite structure with a length of 157,102 bp, which contained inverted repeats (IR) of 26,476 bp separated by a large single-copy (LSC) and a small single copy (SSC) of 85,559 bp and 18,591 bp, respectively. The cpDNA contains 130 genes, comprising 85 protein-coding genes, 37 tRNA genes and 8 rRNA genes. Among the annotated genes, 14 of them contain one intron (*atp*F, *ndh*A, *ndh*B, *rpoC*1, *pet*B, *pet*D, *rpl*16, *rpl*2, *trn*A-UGC, *trn*I-GAU, *trn*G-GCC, *trn*K-UUU, *trn*L-UAA and *trn*V-UAC), and three genes (*rps*12, *clp*P and *ycf*3) contain two introns. The overall GC content of the plastome is 37.3%.

To identify the phylogenetic position of *M. guangxiensis*, phylogenetic analysis was conducted. A neighbor joining (NJ) tree with 1000 bootstrap replicates was inferred using MEGA version 7 (Kumar et al. 2016) from alignments created by the MAFFT (Katoh and Standley 2013) using plastid genomes of 17 species. It showed the position of *M. guangxiensis* is related to the congeneric *Catha edulis* (Figure 1). Our findings can be further used for population genomic and phylogenomic studies of Celastraceae. It will also provide fundamental data for the conservation, utilization and management of this rare species.

**Figure 1.**
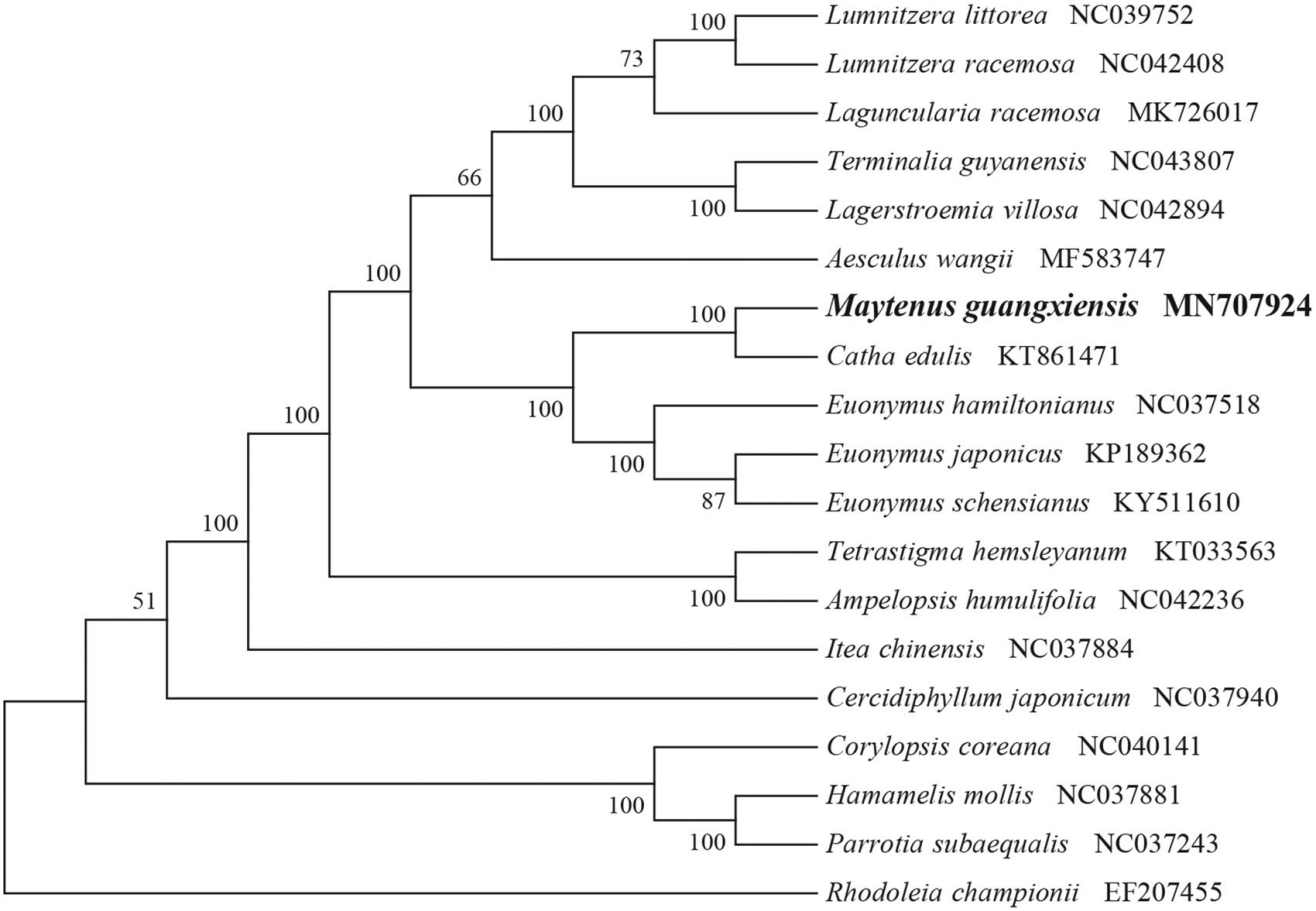
NJ phylogenetic tree of *M. guangxiensis* with 19 species was constructed by chloroplast plastome sequences. Numbers on the nodes are bootstrap values from 1000 replicates. *Rhodoleia championii* was selected as outgroups.
